# Liver cirrhosis and hepatocellular carcinoma attributable to hepatitis B and C in Kyrgyzstan, 2019–2024: a prospective and retrospective study

**DOI:** 10.1016/j.lanepe.2026.101665

**Published:** 2026-03-28

**Authors:** Michael Brandl, Zuridin Nurmatov, Gulsunai Sattarova, Zamira Abdrakhmanova, Saikal Temirbekova, Zhanylai Nuridinova, Timur Dautov, Bekmurza Кanybekov, Beishen Kerimkulov, Indira Akunova, Isa Madanbekov, Sharipa Satybaeva, Elvira Zhakishova, Umut Abdikerim, Zharkynai Torobekova, Ida Sperle, Sandra Dudareva

**Affiliations:** a Department of Infectious Disease Epidemiology, Robert Koch Institute, Berlin, Germany; b Charité – Universitätsmedizin Berlin, Corporate Member of Freie Universität Berlin and Humboldt-Universität zu Berlin, Berlin, Germany; c National Institute of Public Health, Ministry of Health of the Kyrgyz Republic, Bishkek, Kyrgyz Republic; d Osh State University, Osh, Kyrgyz Republic; e National Center of Oncology and Hematology, Bishkek, Kyrgyz Republic; f Jalal-Abad Regional Clinical Hospital, Jalal-Abad, Kyrgyz Republic; g The National Hospital, Ministry of Health of the Kyrgyz Republic, Bishkek, Kyrgyz Republic; h Osh Interregional United Clinical Hospital, Osh, Kyrgyz Republic; i Osh Regional Oncology Center, Osh, Kyrgyz Republic; j Institute of Public Health, Riga Stradins University, Riga, Latvia

**Keywords:** Viral hepatitis, Hepatitis B, Hepatitis C, Hepatitis D, WHO European Region, Central Asia, Kyrgyzstan, Liver cirrhosis, Hepatocellular carcinoma

## Abstract

**Background:**

Hepatitis B, C, and D viruses (HBV/HCV/HDV) can cause chronic infections that lead to sequelae like liver cirrhosis and hepatocellular carcinoma (HCC). We aimed to estimate the fractions of cirrhosis and HCC attributable to HBV and HCV in Kyrgyzstan.

**Methods:**

We collected information on patients diagnosed with cirrhosis and/or HCC from medical records in clinical hospitals and oncology centres in Bishkek, Osh, and Jalalabad. The study included a prospective part from 08/2023 to 04/2024 and a retrospective part from 01/2019 to 07/2023. We calculated attributable fractions (AFs) with 95% confidence intervals (95% CI) for HBV and HCV and stratified results by age, sex, and region. We analysed test results for HBV surface antigen (HBsAg), antibodies to HCV (anti-HCV) and HDV (anti-HDV), and viral DNA/RNA.

**Findings:**

Among participants with cirrhosis, we found AFs of 46% (95% CI 42–49%) for HBV (416/914) and 25% (95% CI 22–28%) for HCV (226/914). Among participants with HCC, AFs to HBV (182/572) and HCV (183/572) were both 32% (95% CI 28–36%). These calculations included 3% of cirrhosis (25/914) and HCC (17/572) patients who had HBV/HCV-coinfections. Among participants tested for anti-HDV, 78% (306/390) of cirrhosis patients and 50% (44/88) of HCC patients tested positive [for anti-HDV].

**Interpretation:**

Large fractions of cirrhosis and HCC cases were attributable to HBV and HCV, with HBV/HDV-coinfections being common. To prevent long-term sequelae and to eliminate viral hepatitis as a public health threat in Kyrgyzstan, earlier testing and treatment options should be implemented.

**Funding:**

This study received financial support in the framework of the Global Health Protection Programme by the Federal Government of Germany.

**Research in context**

**Evidence before this study**

The World Health Organization (WHO) global health sector strategy (GHSS) on viral hepatitis outlines elimination targets to be achieved by the Member States, including reduction of mortality by 65% by 2030. On 27 November 2025, we searched PubMed with search terms (“Hepatitis, Viral, Human” [Mesh] OR “Hepatitis Viruses” [Mesh]) AND (“Liver Cirrhosis” [Mesh] OR “Liver Neoplasms” [Mesh]) AND (“Prevalence” [Mesh] OR “Fraction” OR “Proportion”) AND (“Europe” [Mesh] OR “Asia, Central” [Mesh]) for the years since the publication of the first GHSS in 2016. We found few studies that estimated attributable fractions of cirrhosis and hepatocellular carcinoma (HCC) but none with robust data from the eastern part of the WHO European Region. In 2017, the Global Burden of Disease Study found the highest age-standardized cirrhosis death rate in Central Asia with 39 per 100,000 population but was limited by the poor availability and quality of data. The study estimated that hepatitis C (24.1% among females and 24.0% among males) and hepatitis B (19.0% among females and 23.7% among males) were the second and third most common causes of cirrhosis after alcohol-related liver disease.

**Added value of this study**

To our knowledge, our study is the first to yield systematically analysed national estimates of the fraction of cirrhosis and HCC cases in Kyrgyzstan attributable to HBV, HCV, and HDV infections. With our multicentre design across the three largest cities of Kyrgyzstan, we generated evidence-based data for a high-burden, data-limited setting in the WHO European Region. Our findings provide exact estimates for attributable fractions to HBV and HCV, exceeding previous estimations and those from other countries, and reveal substantially high HBV/HDV coinfections among hepatitis-B-positive patients with cirrhosis and HCC in Kyrgyzstan. This study demonstrates that viral hepatitis accounts for a substantial share of advanced liver sequelae and will inform mortality calculations in Kyrgyzstan.

**Implications of all the available evidence**

All available evidence combined implies, that viral hepatitis infections are dominant drivers of end-stage liver disease in Kyrgyzstan and potentially in further countries in the eastern part of the WHO European region. To track progress toward WHO mortality-reduction targets, periodic repeat studies and continuous surveillance of mortality due to cirrhosis and HCC, as well as the fractions attributable to viral hepatitis, are needed to establish and update national and regional baselines. Member States of the WHO European Region and globally would benefit from deriving national AF inputs to strengthen mortality surveillance and inform national hepatitis elimination strategies.

## Introduction

Mortality due to hepatitis B and C is one of the two major criteria that WHO uses in the Global Health Sector Strategy (GHSS) to define the elimination of viral hepatitis as a public health threat with the target of 65% reduction by 2030.^1^ Untreated, chronic infections with hepatitis B virus (HBV) and hepatitis C virus (HCV) can lead to serious sequelae like liver cirrhosis and hepatocellular carcinoma (HCC).^2^^,^^3^ Hence, one of the essential components of viral hepatitis surveillance is monitoring deaths from cirrhosis and HCC and their underlying causes.^4^

Kyrgyzstan is a landlocked lower-middle-income country in Central Asia with an estimated population of about 7 million people in 2023.^5^ Cirrhosis was the fourth most common cause of death in Kyrgyzstan among both men and women in 2021.^5^ A systematic review, covering the years 1993–2021, found that globally, 42% of patients with cirrhosis had HBV infections and 21% had HCV infections, but lacked data from countries in Central Asia including Kyrgyzstan.^6^ A global analysis of cancer incidence from 2020 named HBV and HCV among the four main oncogenic pathogens and attributed over 500,000 combined annual deaths to these two hepatitis viruses, but evidence from Central Asia was scarce and only one small study from Tajikistan was included in the analysis.^7^ The WHO Global Hepatitis Report 2024 identified Kyrgyzstan as a focus country for the viral hepatitis response in the WHO European Region and underscored that reliable data on progress in control and elimination of HBV and HCV are urgently needed.^8^

According to WHO data, prevalence among the general population of Kyrgyzstan was estimated at 5.3% for chronic infections with HBV and at 2.5% for chronic infections with HCV in 2022.^9^ While newly reported cases of acute viral hepatitis have been steadily declining since the 1990s, the burden of chronic hepatitis infections is increasing in older population groups in Kyrgyzstan.^10^ National estimates of the attributable fraction are essential for understanding the viral hepatis burden, as well as for advocacy, planning, and monitoring of progress towards achieving viral hepatitis targets outlined in the Action Plan for ending the viral hepatitis epidemic in the WHO European Region.^11^

The latest implemented national hepatitis elimination programme for 2023–2027 provided a new opportunity to gain insights on the true burden of HBV and HCV in Kyrgyzstan by offering free testing for hepatitis viruses to large parts of the population.^12^ We aimed to estimate the fraction of cirrhosis and HCC attributable to HBV and HCV in Kyrgyzstan. By combining prospective data collection with retrospective examination of medical records, we intended to assess validity of retrospectively acquired data. Additionally, we describe clinical and demographic characteristics of cirrhosis and HCC patients in Kyrgyzstan to inform public health action for reducing mortality due to long-term consequences of viral hepatitis.

## Methods

### Study population

Our target population was patients aged 18 years or older with a diagnosis of cirrhosis and/or HCC in Kyrgyzstan who were registered as patients at the participating study sites from 2019 onwards. We excluded participants with neither cirrhosis nor HCC diagnosis and participants with no information on hepatitis testing.

### Study sites

The study took place in the following six study sites across the three largest cities of Kyrgyzstan: Bishkek (Gastroenterology Department of the National Hospital, National Centre for Oncology and Haematology with Polyclinic), Osh (Osh Interregional United Clinical Hospital, Osh Interregional Oncology Centre), and Jalal-Abad (Regional Clinical Hospital: Therapy Department, Oncology Department). These healthcare facilities represent predominantly tertiary-care hospitals in Kyrgyzstan.

### Study design

The study included a prospective and a retrospective study part. All patients, who presented with cirrhosis and/or HCC at one of the participating study sites during the prospective study period (August 2023–April 2024), were informed about the study and included, unless they opted out. Blood samples were drawn and tested for hepatitis B surface antigen (HBsAg) and antibodies against HCV (anti-HCV) in the course of the regular medical examination. If tested positive for HBsAg, testing for antibodies against HDV (anti-HDV) was performed. Wherever possible, samples positive for HBsAg, anti-HCV, and anti-HDV were subsequently tested for HBV DNA, HCV RNA, and HDV RNA, respectively. Laboratory testing was performed at the National Institute of Public Health (NIPH) or the Republican Center for AIDS. All testing costs were covered by the national hepatitis elimination programme.

In the retrospective part of the study, patients diagnosed with cirrhosis in the period between January 2022 and July 2023 and patients diagnosed with HCC between January 2019 and July 2023 were identified through review of medical records and included in the study. Information on diagnoses with hepatitis B and C, as well as additional information on testing results for hepatitis seromarkers, demographics, treatment, and individual risk factors were extracted. Retrospectively, subsequent PCR testing was not routinely available, as testing capacities were limited and PCR testing generally associated with costs for patients.

To calculate the sample size, we used an expected attributable fraction of 50% based on expert opinion and unpublished results of previous investigations of roughly 250 cirrhosis patients at the NIPH in Kyrgyzstan. We set the precision to 5% and used 95% confidence intervals (95% CI), resulting in a total sample size of 385 participants. To account for potential errors in medical records and non-response in the prospective study part, we set the target sample size to 400 cases of cirrhosis and 400 cases of HCC. This sample size allowed for the calculation of separate attributable fractions for HBV and HCV.

### Data collection

Trained investigators from Kyrgyzstan conducted the data extraction by reviewing medical records and entered the data electronically, using the offline data collection software EpiData. Data was retrieved according to a case extraction form which was based on the WHO's protocol for surveillance of the fraction of cirrhosis and HCC attributable to viral hepatitis in clinical centres of excellence.^4^ We adapted the WHO protocol to reflect medical practices and availability of data in Kyrgyzstan and provide an English translation of the form in Supplementary Material, Table S1.

From medical records of patients, we collected information on diagnoses with cirrhosis (ICD-10 K74) and/or HCC (ICD-10C22.0). For cirrhosis, we collected information if the disease was diagnosed for the first time, the cirrhosis stage by Child-Pugh-Score (Child A: 5–6 points, Child B: 7–9 points, Child C: 10–15 points), and the disease outcome (monitoring, transplantation, death). We extracted demographic information on age at the time of the medical examination (age groups: <41, 41–50, 51–60, 61–70, >70 years), sex, study site, and place of residence (7 regions and 2 independent cities of Kyrgyzstan). No personal identifiable data from patients were collected during the data extraction from the medical records.

Where available, we additionally collected the most recent test results of hepatitis infection seromarkers: HBsAg and HBV DNA for HBV infection, anti-HCV and HCV RNA for HCV infection, and anti-HDV and HDV RNA for HDV infection. Additionally, we collected information on medical treatment (treatment received, treatment regimen, month of treatment initiation for HCV) and individual risk factors for liver cirrhosis and HCC (alcohol use, hypertension, steatotic liver, diabetes, obesity (BMI > 30), autoimmune hepatitis, other risk factor).

### Data analysis

We described participants with cirrhosis and HCC by age group, sex, and study site and report numbers and proportions with 95% CI. We compared differences between the two study parts in regards to age groups and sex using Pearson's Chi-Squared test. Participants diagnosed with both cirrhosis and HCC were analysed as HCC cases following protocols developed by the European Centre for Disease Prevention and Control (ECDC) and WHO for three pilot studies in Europe.^13^ Additionally, we described numbers of participants by place of residence on regional level by absolute values and relative to the population size of each region per 100,000 population.

We grouped participants into four viral hepatitis outcome groups: hepatitis B diagnosis only, hepatitis C diagnosis only, both hepatitis B and C diagnosis, no viral hepatitis diagnosis. We report frequencies and proportions for each of these outcome groups, stratified by study part, with 95% CI. We checked for statistical differences in the outcome groups between study parts using Pearson's Chi-Squared test.

We assessed data completeness for all above mentioned variables. No systematic missing data were observed across study sites and missing observations are reported in the tables. Where results on specific seromarkers were available, we report numbers of overall performed tests and proportions positive and negative.

For both cirrhosis and HCC patients, we report frequencies of hepatitis B and C diagnosis and attributable fractions (AFs) with 95% CI. We calculated 95% CI using the Wilson score method. We stratified results by age group, sex, and region of residence. Generally, AF is calculated using the formula $\text{AF}=p_{e}\;\frac{\left(\text{RR}-1\right)}{\;\text{RR}}$ from the risk ratio (RR) and the proportion exposed ($p_{e}$). As RR is very high for infections with HBV and HCV (RR > 10), AF can be approximated as $p_{e}$ to HBV and HCV among cirrhosis and HCC patients and the general formula can be simplified to $\text{AF}=p_{e}$.^13^^,^^14^ Participants with HBV/HCV coinfection were included in the individual calculations of AFs for both HBV and HCV.

We report overall numbers of patients with cirrhosis and HCC on treatment and of most commonly prescribed medication regimens for hepatitis B (Tenofovir, Entecavir, Interferon) and hepatitis C (Sofosbuvir in combination with Velpatasvir, Daclatasvir, or Ledipasvir). For HCV treatment, we calculated the time difference in months from treatment initiation to inclusion in the study (i.e. date of the medical record) to identify participants who were already successfully treated for an HCV infection. We assumed a maximum treatment duration of 12 weeks for direct-acting antivirals (DAAs), followed by 12 weeks for the assessment of sustained virologic response (SVR12) after treatment end. Based on this assumption, we stratified HCV RNA results by whether treatment was initiated more or less than six months prior to study inclusion.

We used UpSet plots for quantitative visualisation of recorded risk factors including hepatitis B and C diagnosis.^15^ In these plots, sets represent total numbers of participants with a specific risk factor, while intersections represent combinations of one or more of these factors. We report the 20 most common intersections for cirrhosis and HCC, respectively.

In sub-group analysis, we used mixed-effects multivariable logistic regression to assess associations between diagnosis with both cirrhosis and HCC compared to cirrhosis alone. We included hepatitis B, hepatitis C, age, sex, and individual risk factors as independent variables in the model and study centres as random intercept to account for clustering. We report adjusted Odds Ratios (aOR) with 95% CI.

All statistical analyses were performed with R (version 4.5.1).

### Ethics approval

The study protocol was reviewed and approved by the Ethics Committee of the Scientific and Production Association ‘Preventive Medicine’ in the Ministry of Health of the Kyrgyz Republic on 11 July 2023 (protocol № 11). The requirement for informed consent was waived by the ethics committee due to the observational nature of the study.

### Role of the funding source

The funders had no role in study design, data collection, analysis, or interpretation, writing of the report, or decision to submit for publication.

## Results

### Study population

From a total of 1489 participants in the study, we excluded 3 (0.2%) participants (2 for missing information on hepatitis testing and 1 for missing diagnosis of cirrhosis or HCC). This led to an analytical sample of 1486 participants, 914 diagnosed with cirrhosis and 572 with HCC. Detailed information on demographic characteristics of the study population with a comparison of the two study parts, as well as a flowchart are available in Supplementary Material, Table S2 and Figure S1. Data on diagnosis, staging, and outcome for cirrhosis and HCC patients are provided in Supplementary Material, Table S3.

**Fig. 1:**
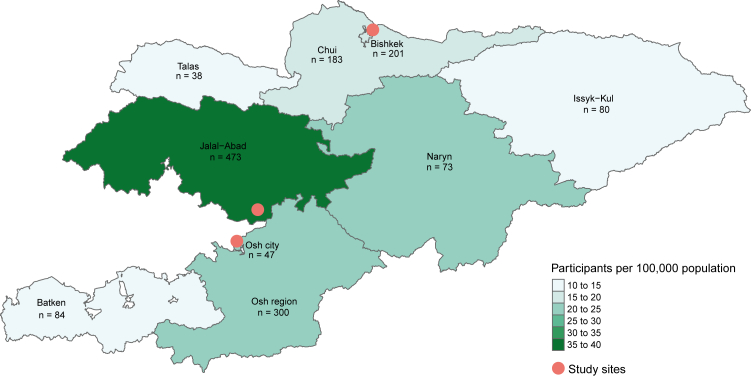
**Absolute numbers (n) and relative numbers (per 100,000 population) of participants by place of residence, Kyrgyzstan, 2024**.

Overall, most participants by place of residence by absolute numbers came from Jalal-Abad (32%, 473/1486), followed by Osh region (20%, 300/1486), and Bishkek (14%, 201/1486). Least participants were from Talas (3%, 38/1486), Osh city (3%, 47/1486) and Naryn (5%, 73/1486). In relative terms, most participants were from Jalal-Abad (35 per 100,000 population), Naryn (23 per 100,000), and Osh region (20 per 100,000). Least participants relative to resident population came from Osh city (13 per 100,000), followed by Talas and Batken (both 14 per 100,000) and Issyk-Kul (15 per 100,000). Absolute and relative numbers of participants per region are displayed in Fig. 1.

### Hepatitis B and C diagnosis

**Fig. 2:**
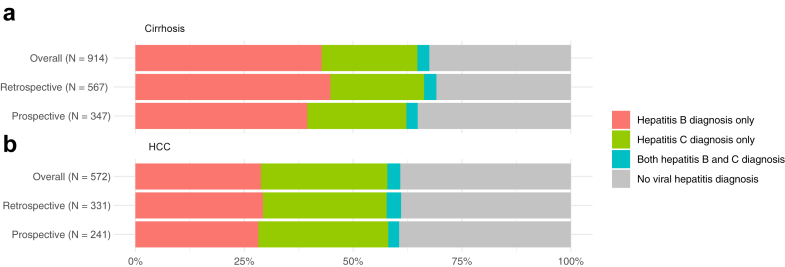
**Numbers (N) of participants with a) cirrhosis and b) hepatocellular carcinoma (HCC) and proportions of participants diagnosed with hepatitis B, hepatitis C, or both hepatitis B and C, stratified by study part, Kyrgyzstan, 2024**.

Among participants with cirrhosis, 43% (391/914) were diagnosed with hepatitis B and 22% (201/914) with hepatitis C. Among HCC patients, 29% (165/572) were diagnosed with hepatitis B and 29% (166/572) with hepatitis C. Three percent of cirrhosis patients (25/914) and of HCC patients (17/572) were diagnosed with both hepatitis B and C. Results of outcome groups stratified by study part are displayed in Fig. 2. We compared the results of the four outcome groups in more detail in Supplementary Material, Table S4, and considered findings across study parts to be sufficiently comparable to continue analyses with the pooled dataset.

### Seromarker results

**Fig. 3:**
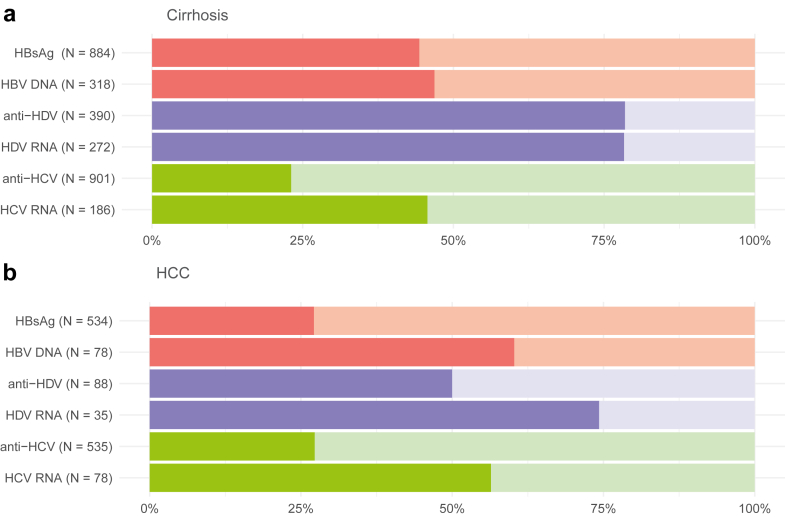
**Numbers (N) of performed tests and percentages (%) of positive and negative test results for hepatitis B, D, and C seromarkers among participants with a) cirrhosis and b) hepatocellular carcinoma (HCC), Kyrgyzstan, 2024**. Only participants with information on respective seromarker tests were included. Darker color shades for positive, lighter color shades for negative test results. HBsAg: hepatitis B surface antigen, HBV DNA: hepatitis B virus DNA, anti-HDV: antibodies against hepatitis D virus, HDV RNA: hepatitis D virus RNA, anti-HCV: antibodies against hepatitis C virus, HCV RNA: hepatitis C virus RNA.

For the majority of hepatitis-positive cases, information on tested seromarkers was available (hepatitis B: 543/598, hepatitis C: 364/409). Fig. 3 shows numbers of performed tests and percentages of participants who had information on performed tests for hepatitis B, D, and C seromarkers. Not all participants were tested for all possible seromarkers, therefore numbers of tests differ by seromarker.

Among cirrhosis participants, the majority positive for HBsAg was also tested for HBV DNA with about half of them testing positive (47%, 149/318). Even more HBsAg-positive participants were tested for anti-HDV with most testing positive (78%, 306/390). Of those consecutively tested for HDV RNA, also 78% were positive (213/272). Cirrhosis participants, who tested positive for anti-HCV and were then tested for HCV RNA, were found to be 46% positive (85/186).

For participants with HCC, those positive for HBsAg and then tested for HBV DNA made out 60% (47/78). The proportion of anti-HDV positivity among HBsAg-positive participants with HCC was lower compared to participants with cirrhosis (50%, 44/88). The positivity for HDV RNA among anti-HDV-positive participants was 74% (26/35). More than half of HCC participants positive for anti-HCV also tested positive for HCV RNA (56%, 44/78).

### Attributable fractions

We found an overall AF for HBV among cirrhosis patients of 46% (95% CI 42–49%). Highest AFs were found among participants <41 years of age (66%, 95% CI 59–73%), male participants (50%, 95% CI 46–55%), and participants from Batken (64%, 95% CI 51–76%) and Jalal-Abad (56%, 95% CI 51–61%). The AF for HCV in cirrhosis patients was overall 25% (95% CI 22–28%) and higher in 61–70-year-old participants (37%, 95% CI 30–45%) and in Osh City (54%, 95% CI 38–70%) (Table 1).

**Table 1:** Numbers (n) of participants with cirrhosis and hepatocellular carcinoma (HCC) and attributable fractions (AF in % with 95% confidence intervals, 95%CI) for hepatitis B and C, stratified by age group, sex, and region, Kyrgyzstan, 2024.

Cirrhosis	Hepatitis B		Hepatitis C		Overall
	n	AF % (95% CI)	n	AF % (95% CI)	N
**Age at medical examination**					
<41	109	66 (59–73)	18	11 (7–17)	164
41–50	143	55 (49–60)	45	17 (13–22)	262
51–60	110	40 (34–46)	87	31 (26–37)	277
61–70	46	29 (22–36)	60	37 (30–45)	161
>70	8	16 (8–29)	16	32 (21–46)	50
**Sex**					
Female	187	41 (36–45)	119	26 (22–30)	458
Male	228	50 (46–55)	107	24 (20–28)	455
Missing	1				1
**Region**					
Bishkek	38	36 (27–45)	36	34 (25–43)	107
Osh City	14	40 (26–56)	19	54 (38–70)	35
Batken	36	64 (51–76)	13	23 (14–36)	56
Osh Region	62	44 (36–52)	41	29 (22–37)	141
Jalal-Abad	184	56 (51–61)	50	15 (12–19)	329
Talas	11	42 (26–61)	5	19 (9–38)	26
Naryn	23	53 (39–67)	7	16 (8–30)	43
Issyk-Kul	11	19 (11–31)	13	23 (14–35)	57
Chui	36	31 (23–39)	41	35 (27–44)	118
Missing	1		1		2
**Total**	**416**	**46 (42–49)**	**226**	**25 (22–28)**	**914**

HCC	Hepatitis B		Hepatitis C		Overall
	n	AF % (95% CI)	n	AF % (95% CI)	N
**Age at medical examination**					
<41	5	31 (14–56)	5	31 (14–56)	16
41–50	24	37 (26–49)	21	32 (22–44)	65
51–60	59	41 (33–49)	50	35 (27–43)	144
61–70	80	31 (25–37)	83	32 (27–38)	260
>70	14	16 (10–25)	24	28 (19–38)	87
**Sex**					
Female	46	23 (18–29)	75	37 (31–44)	201
Male	136	37 (32–42)	108	29 (25–34)	371
**Region**					
Bishkek	22	23 (16–33)	30	32 (23–42)	94
Osh City	6	50 (25–75)	3	25 (9–53)	12
Batken	9	32 (18–51)	10	36 (21–54)	28
Osh Region	67	42 (35–50)	54	34 (27–42)	159
Jalal-Abad	45	31 (24–39)	55	38 (31–46)	144
Talas	6	50 (25–75)	1	8 (1–35)	12
Naryn	5	17 (7–34)	6	20 (10–37)	30
Issyk-Kul	5	22 (10–42)	7	30 (16–51)	23
Chui	16	25 (16–36)	16	25 (16–36)	65
Missing	1		1		5
**Total**	**182**	**32 (28–36)**	**183**	**32 (28–36)**	**572**

Among patients with HCC, we found an AF for HBV of 32% (95% CI 28–36%), which was higher among male (37%, 95% CI 32–42%) than among female participants (23%, 95% CI 18–29%). The AF for HCV among HCC patients was equally high with 32% (95% CI 28–36%), but confidence intervals for all demographic and regional strata overlapped.

### Treatment

Among participants with cirrhosis and viral hepatitis infections, 64% (243/416) received treatment for HBV infections and 69% (142/226) for HCV infections. Among HCC patients with viral hepatitis, 26% (36/182) received hepatitis B treatment and 30% (46/183) hepatitis C treatment. Treatment regimens are provided in Supplementary Material, Table S5.

Of the participants for whom information on hepatitis C treatment initiation was available, 54% (92/170) had started treatment at least six months before being included in the study. Among participants with additional information on HCV RNA testing, 13% (11/86) tested positive for HCV RNA when treatment initiation had been six or more months ago, compared to 84% (47/56) of participants with more recent treatment initiation.

### Risk factors

**Fig. 4:**
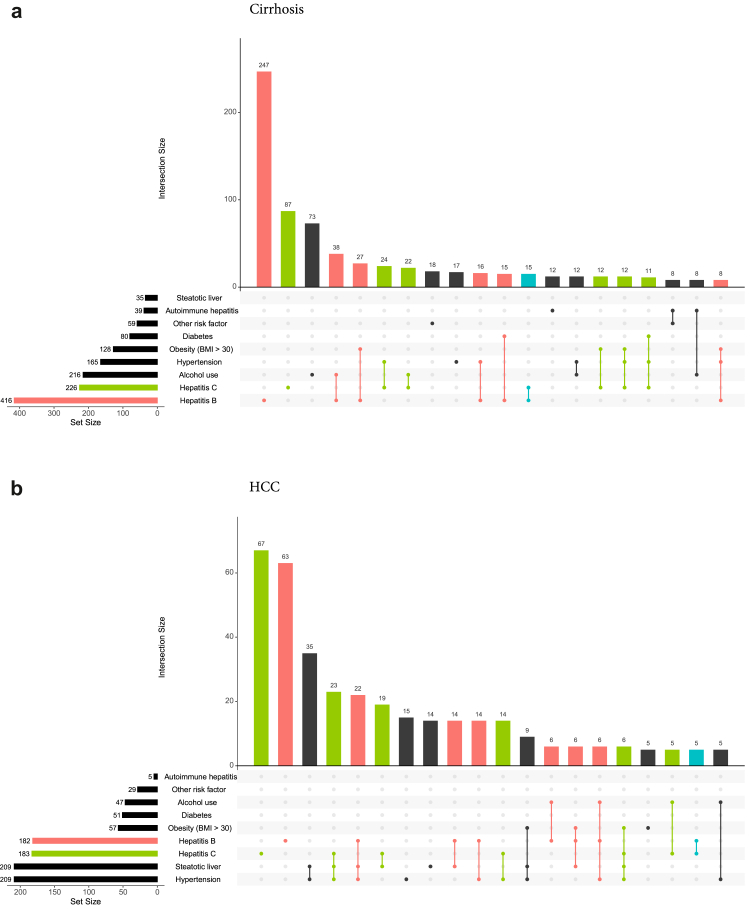
**UpSet plots depicting the frequencies and intersections of viral hepatitis infections and individual risk factors among participants with a) cirrhosis and b) hepatocellular carcinoma (HCC), Kyrgyzstan, 2024**. Horizontal bars show set sizes, i.e. overall numbers of participants with the recorded risk factors. Vertical bars show intersection sizes, i.e. numbers of participants who reported specific combinations of one or more risk factors, as indicated by the dot matrix below. Red color: set for hepatitis B and all intersections including hepatitis B. Green color: set for hepatitis C and all intersections including hepatitis C. Blue color: intersection for both hepatitis B and C.

Among cirrhosis patients, alcohol use was overall the third most commonly reported risk factor with 24% (216/914), after hepatitis B (46%, 416/914) and hepatitis C (25%, 226/914) (Fig. 4a). These three risk factors were also most commonly reported as sole risk factors in cirrhosis patients with 27% (247/914) for hepatitis B, 10% (87/914) for hepatitis C, and 8% (73/914) for alcohol use. For a total of 9% (82/914) of cirrhosis patients, neither hepatitis infections nor any individual risk factors were recorded.

For HCC patients, hypertension and steatotic liver were overall the most commonly reported risk factors with 37% (209/572) each, followed by hepatitis C (32%, 183/572) and hepatitis B (32%, 182/572) (Fig. 4b). Hepatitis C (12%, 67/572) and hepatitis B (11%, 63/572) were most commonly recorded as sole risk factors, followed by the intersection of hypertension and steatotic liver (6%, 35/572). For 19% (106/572) of HCC patients there was no medically recorded individual risk factor or hepatitis infection.

In multivariable logistic regression adjusting for infections with both hepatitis viruses, demographic variables, and individual risk factors, HCC diagnosis among cirrhosis patients was positively associated with hepatitis B (aOR = 2.4, 95% CI 1.2–5.0), male sex (aOR = 3.8, 95% CI 1.9–7.4), and higher age (aOR = 3.0, 95% CI 1.5–5.8, age group 61–70 vs age group 51–60) (Supplementary Material, Table S6).

## Discussion

Our study was the first analysis of the AF for HBV and HCV among patients with cirrhosis and HCC in Kyrgyzstan. Large proportions of participants had been diagnosed with hepatitis B or C and coinfections with HBV and HDV were very common, especially among cirrhosis patients. While the study population is not representative of the Kyrgyz general population, this suggests a high estimated prevalence of chronic infections with HBV and HCV in higher age groups. The prospective and retrospective study parts yielded comparable estimates, indicating internal consistency of our results across study parts and validity of calculating AFs based on retrospective data from medical records.

Among cirrhosis patients, we found a higher fraction attributable to hepatitis B than hepatitis C, which is in line with the generally higher burden of hepatitis B in Kyrgyzstan.^9^ Chronic HBV infections remain uncurable and only lifelong medication can control the virus. While the overall numbers of participants with cirrhosis in younger age groups were low, we found higher AFs due to HBV among them. They may have been infected during birth or in early childhood, as HBV infections at young age often stay asymptomatic but persist for many years and liver diseases including cirrhosis can develop up to 40 years later.^16^

Most HBsAg-positive participants were also tested for anti-HDV, and the majority of patients with cirrhosis and about half of patients with HCC tested positive. Chronic infections with HDV are considered the most aggressive form of viral hepatitis and the infection accelerates progression to late-stage sequalae.^17^ A systematic review from 2020 estimated a global anti-HDV prevalence of 16.4% (14.6–18.6%) among HBsAg-positive patients at healthcare facilities and that HDV is responsible for 18% of cirrhosis and 20% of HCC cases among patients with hepatitis B.^18^ The prevalence in Central Asia is considered higher and our results even exceed recent estimations of 42% anti-HDV prevalence in HBsAg-positive cirrhosis patients in Kyrgyzstan.^19^

Vaccination with three doses of hepatitis B vaccine, best administered in early childhood, offers very long to possibly lifelong protection.^20^ The universal vaccination programme in Kyrgyzstan was successfully implemented in 1999, reaching high hepatitis B vaccination coverage from 2002.^21^ A serosurvey conducted in 2022 showed very low prevalence of 0.12% (95% CI 0.04–0.35%) among targeted birth cohorts.^22^ Consequently, our study estimations for cirrhosis and HCC largely reflect HBV infections acquired prior to the introduction of universal vaccination. In vaccinated cohorts, we expect the burden of both cirrhosis and HCC to substantially decrease, as HBV will no longer contribute as an etiological factor.

For HCV and cirrhosis, we found the inverse relationship and higher proportions positive among older participants, which is line with other studies that found higher anti-HCV prevalence estimates among older age cohorts.^23^ Chronic hepatitis C is usually slowly progressing and development of cirrhosis can take 20–30 years.^24^ With increasing age, the cumulative risk of exposure to HCV rises, especially in settings where unsafe medical procedures or transfusions were common in the past. As chronic HCV infections are often asymptomatic for decades, many individuals remain undiagnosed and their disease may progress to cirrhosis or HCC.

Among participants with HCC, we found similar AFs for HBV and HCV of 32%, which is in line with previous studies: a statistical modelling study, published in 2018 and using global data from 2012, estimated the prevalence of liver cancer in Kyrgyzstan attributable to HBV at 44% (10–72%) and to HCV at 32% (3–78%).^25^ The very high uncertainty in these models was due to a lack of national prevalence data of HBV and HCV infections in HCC patients from Central Asian countries, a gap that our study aims to fill for Kyrgyzstan. While the overall impact of the national testing programme on the hepatitis burden in Kyrgyzstan has not yet been evaluated, its outcomes in combination with our study results could facilitate monitoring of risk factors for liver disease, including not only viral hepatitis infections, but also other risk factors like alcohol consumption and metabolic dysfunction-associated steatotic liver disease (MASLD). Future studies in Kyrgyzstan should aim to quantify the programme's impact in order to guide public health planning and resource allocation. Continued implementation of the testing programme could support earlier detection of liver disease and prevent progression to cirrhosis and HCC.

While for HCV infections, the sex ratio was quite equally distributed, male cirrhosis and HCC patients were more likely to be diagnosed with hepatitis B than female patients. We hypothesize that early detection may be a larger issue among men in Kyrgyzstan, as women have more frequent contact with the healthcare system in general and for antenatal care during pregnancies, which could lead to hepatitis B diagnosis before cirrhosis develops. Future generations may be protected from infections with HBV regardless of sex through universal vaccination, but the high AF for HBV among cirrhosis and HCC patients highlights that adults in Kyrgyzstan should get screened for viral hepatitis and in case positive be linked to treatment. Additional capacity building in the healthcare workforce of Kyrgyzstan may be necessary to increase uptake of free testing offers. A mixed-methods study conducted in 2024 among medical doctors working in primary healthcare facilities in Kyrgyzstan identified lack of training and time, limited work force, and stigma and discrimination towards key populations as main barriers for scaling-up viral hepatitis testing.^26^

Most participants both in absolute and relative terms by place of residence were from region Jalal-Abad and doctors from Kyrgyzstan suggested that liver conditions appear to be more common there. Participants with cirrhosis from Jalal-Abad had an above average AF for HBV but a below average AF for HCV. While our study was not powered or designed to identify regional differences in cirrhosis and HCC prevalence, this finding may hint at increased need for tailored public health measures in certain regions of Kyrgyzstan. Ongoing mortality surveillance of cirrhosis and HCC throughout the country may help identify specific risk factors across regions of Kyrgyzstan and highlight potential for prevention.

Most HCC patients were already in late stages of their disease and less than one in three received treatment for the underlying HBV or HCV infections. This highlights missed opportunities for earlier detection of viral hepatitis infections and prevention of HCC as long-term consequence. DAAs have been available since 2011 and shown to drastically reduce the risk of HCC development.^27^ In a mathematical modelling study on prevention and treatment of hepatitis C, including outreach screening and offering DAAs at hepatitis diagnosis, Kyrgyzstan could largely reduce chronic infections and HCV-related deaths.^28^ The national hepatitis elimination plan of Kyrgyzstan for 2023–2027 allocated funds to reimburse treatment of 10,000 patients with hepatitis B and 5000 patients with hepatitis C, making treatment available for many patients outside of private pharmacies for the first time.^12^ In the Global Hepatitis Report, WHO estimated that the overall treatment coverage in Kyrgyzstan was only 0.3% for hepatitis B and 4% for hepatitis C in 2022.^8^ In our study, the proportions of participants who received treatment was notably higher, especially among cirrhosis patients, likely due to increased contact with the healthcare system and treatment in tertiary-care hospitals. While participants with advanced stages of liver disease may be overrepresented in our sample and broader testing of the Kyrgyz population could reduce AFs for HBV and HCV, there is undeniably still a large part of the population in need for treatment.

Only parts of HBsAg- and anti-HCV-positive participants were consequently tested with PCR for presence of viral DNA or RNA. Under the new Kyrgyz hepatitis elimination programme, not only screening for antibodies and antigen, but also PCR testing is free for patients and we observed higher PCR testing rates in the prospective study part (data not shown). About half of the patients with hepatitis B and C and the majority of patients with hepatitis D who underwent PCR testing, were found to be positive. For hepatitis C, our results suggest that the majority of participants with negative HCV RNA results had successfully been treated with DAAs. Their diagnoses with cirrhosis or HCC however highlight that even after sustained virologic response, a residual elevated risk for development of liver disease remains, especially in the presence of additional risk factors.^29^ Facilitation of testing, subsequent referral to treatment, and prolonged monitoring for both hepatitis B and C will be key to reduce transmission and prevent long-term consequences like cirrhosis and HCC.^30^^,^^31^

Infections with hepatitis viruses, especially HBV, were reported as the single most common risk factors among both cirrhosis and HCC patients, which underscores the strong link between hepatitis infections and development of late-stage sequalae. Alcohol use was the most commonly reported individual risk factor and studies have shown that even in smaller doses, it can lead to fibrosis and steatotic liver disease or aggravate the course of a viral hepatitis infection, leading WHO to recommend a zero-alcohol policy.^32^^,^^33^ Of the five Central Asian countries, Kyrgyzstan ranked first in regards to the fractions of cirrhosis deaths due to alcohol consumption with an estimated 54% in 2019 among the population aged 15 and older, highlighting the enormous potential reductions in alcohol consumption could add in reduction of cirrhosis incidence.^34^ Among participants with HCC, chronic infections with HBV and HCV have also been recorded as main independent risk factors, but combinations of metabolic risk factors like steatotic liver and hypertension were also frequent. MASLD causes chronic inflammation and fibrogenesis, which can contribute to HCC development in patients both with and without cirrhosis.^35^ Our study was not designed to quantify independent effects of multiple risk factors, nevertheless in sub-group analyses we found that infections with HBV, as well as male sex and higher age were independently associated with HCC diagnosis among cirrhosis patients. Therefore, adults in Kyrgyzstan born before the introduction of hepatitis B vaccination represent an important target group for interventions aimed at prevention of late-stage liver disease.

Our study has several limitations. With our study design, we cannot draw causal conclusions between exposures like hepatitis infections or other risk factors and the outcomes cirrhosis and HCC. Our sample includes patients from the entire country but is not representative of the population of Kyrgyzstan and overrepresents participants from Jalal-Abad. Patients likely travelled to the large tertiary-care hospitals, which were our study centres, leading to participants from further remote regions Batken, Issyk-Kul, and Talas being underrepresented. This is especially true for the capital Bishkek, as it represents Kyrgyzstan's main centre for specialised care in hepatology and liver oncology and patients were referred there from primary and secondary healthcare providers. As our study was primarily conducted in tertiary-care facilities, which is reflected by the low proportion of cirrhotic patients in Child stadium A, our study population does not reflect the overall population structure of liver cirrhosis in Kyrgyzstan. While our target sample size for the overall AF was reached, our study was not powered to quantify differences across demographic and regional strata or the independent effects of multiple risk factors in logistic regression. While the high concordance between prospective and retrospective study results suggests internal validity, we cannot rule out systematic errors. Selection bias could have occurred during the retrospective study period, as access to free-of-charge testing was very limited. Patients who could afford to get tested for HBV and HCV infections could differ systematically from those who could not and viral hepatitis infections may be substantially underdiagnosed among cirrhosis and HCC patients in the Kyrgyz population. The high proportion of participants who received hepatitis B and C treatment might indicate a preselection of participants with viral hepatitis infections. Additionally, retrospective data collection is prone to information bias and in case negative testing results were not documented or retrieved from medical records, this could have led to an overestimation of AFs. Our approximations to asses SVR12 for HCV treatment may have led to misclassification of treatment status and consequent interpretation of HCV RNA test results for some participants. Risk factors like alcohol use, which were documented by doctors in medical records, were often based on a subjective assessment.

We found large fractions of cirrhosis and HCC attributable to infections with HBV, often with superinfection of HDV, and HCV. Our study demonstrates huge gaps in timely testing and treatment of viral hepatitis and highlights the need for improved surveillance of infections and related mortality in Kyrgyzstan. The recently successfully implemented national testing programme offers new opportunities for earlier detection of viral hepatitis infections and prevention of late-stage liver diseases like cirrhosis and HCC. To measure progress towards WHO mortality reduction targets, it will be key to carry out continuous monitoring and repeat studies. In limited-resource settings, retrospective data collection can provide robust results, as long as strict adherence to the study methodology is guaranteed. Further countries in the WHO European Region and worldwide should feel encouraged to assess the attributable fraction of hepatitis B and C among cirrhosis and HCC cases and thereby generate evidence for the path to viral hepatitis elimination based on robust national data.

## Contributors

Conceptualization: MB, ZN, IS, SD; Data curation: MB, GS, ZA, ST, ZhN, TD; Formal analysis: MB; Investigation: BK, BKe, IA, IM, SS, EZ, UA, ZT; Methodology: MB, IS, SD; Supervision: ZN, SD; Validation: MB, SD; Visualisation: MB; Writing—Original Draft: MB; Writing—Review & Editing: all authors. MB and SD assessed and verified the data. All authors approved the final version of the manuscript and accept responsibility for the decision to submit the manuscript for publication.

## Data sharing statement

With publication, the data that support the findings of this study are available from the corresponding author upon reasonable request.

## Editor note

The Lancet Group takes a neutral position with respect to territorial claims in published maps.

## Declaration of interests

All authors declare no competing interests.

## Appendix A Supplementary data

Supplementary Material

AF_translated_abstract_Russian_notitle
