# Supplementary material for: Liver cirrhosis and hepatocellular carcinoma attributable to hepatitis B and C in Kyrgyzstan, 2019–2024: a prospective and retrospective study
Source: Lancet Reg Health Eur. 2026 Mar 28;65:101665. doi: 10.1016/j.lanepe.2026.101665 (PMC13062556; doi:10.1016/j.lanepe.2026.101665)
Supplement: Supplementary Material [file mmc1.pdf]

## Supplementary Material to:

# Liver cirrhosis and hepatocellular carcinoma attributable to hepatitis B and C in Kyrgyzstan, 2019–2024: a prospective and retrospective study


Table S1: Data collection form, translated to English ........ 2

Table S2: Characteristics of participants with cirrhosis and HCC regarding age, sex, and study site, stratified by study part, Kyrgyzstan, 2024 (n = 1,486) ........ 4

Figure S1: Study flowchart showing the composition of the study population and excluded participants ........ 5

Table S3: Characteristics of participants with cirrhosis and HCC regarding diagnosis, staging, and outcome, Kyrgyzstan, 2024 (n = 1,486) ........ 6

Table S4: Hepatitis diagnosis among participants with cirrhosis and HCC, stratified by study part, Kyrgyzstan, 2024 (n = 1,486) ........ 7

Table S5: Hepatitis B and C treatment regimens of participants with cirrhosis and HCC, Kyrgyzstan, 2024 (n = 427) ........ 8

Table S6: Results of mixed-effects multivariable logistic regression with outcome HCC diagnosis among cirrhosis patients, including study sites as random intercept, Kyrgyzstan, 2024 (n = 1,188).. 9

## Table S1: Data collection form, translated to English

### 1. General characteristics

| | |
|---|---|
| Study part | ☐ 1 – Retrospective<br>☐ 2 – Prospective |
| Medical Centre | ☐ 1 – Bishkek: Gastroenterology Department of the National Hospital<br>☐ 2 – Bishkek: National Centre for Oncology and Haematology with Polyclinic<br>☐ 3 – Osh: Osh Interregional United Clinical Hospital<br>☐ 4 – Osh: Osh Interregional Oncology Centre<br>☐ 5 – Jalal-Abad: Regional Clinical Hospital: Therapy Department<br>☐ 6 – Jalal-Abad: Regional Clinical Hospital: Oncology Department |
| ID number (xxx) | |
| Date of completion of medical record (dd/mm/yyyy) | |
| Date of birth (dd/mm/yyyy) | |
| Age (years) | |
| Sex | ☐ Male<br>☐ Female |
| Place of residence | |

### 2. Patient with liver cirrhosis

| | |
|---|---|
| Liver cirrhosis | ☐ Yes<br>☐ No<br>☐ Unknown (If no or unknown, proceed to section 3) |
| First-time diagnosis | ☐ Yes<br>☐ No<br>☐ Unknown |
| Cirrhosis stage (according to Child-Pugh) | ☐ A (5-6 points)<br>☐ B (7-9 points)<br>☐ C (10-15 points)<br>☐ Unknown |
| Outcome | ☐ Observation<br>☐ Transplantation<br>☐ Death<br>☐ Unknown |

### 3. Patient with hepatocellular carcinoma (HCC)

| | |
|---|---|
| HCC | ☐ Yes<br>☐ No<br>☐ Unknown (If no or unknown, proceed to section 4) |
| First-time diagnosis | ☐ Yes<br>☐ No<br>☐ Unknown |
| Diagnostic method (check all that apply) | ☐ Clinical/laboratory<br>☐ Instrumental<br>☐ Pathological<br>☐ Unknown |
| HCC Stage*[3] | ☐ I<br>☐ II<br>☐ III<br>☐ IV<br>☐ Unknown |
| Outcome | ☐ Observation<br>☐ Transplantation<br>☐ Death<br>☐ Unknown |

### 4. Diagnosis of hepatitis B virus (HBV)

| | |
|---|---|
| HBV diagnosis | ☐ Positive, and serological marker results are available<br>☐ Positive, but no serological marker results available<br>☐ Negative<br>☐ Unknown (If unknown, proceed to section 5) |
| HBsAg | ☐ Positive<br>☐ Negative<br>☐ Not performed<br>☐ Unknown |

| | | | |
|---|---|---|---|
| HBV DNA | ☐ Positive<br>☐ Negative<br>☐ Not performed<br>☐ Unknown | | |
| HBV DNA quantity | Copies/ml | IU/ml | log IU/ml |
| Currently undergoing treatment for HBV | ☐ Yes<br>☐ No<br>☐ Unknown | | |
| If yes, treatment regimen | ☐ Tenofovir (TDF and TAF)<br>☐ Entecavir<br>☐ Interferon (INF)<br>☐ Other<br>☐ Unknown | | |
| If other treatment, please specify: | | | |
| **Diagnosis of hepatitis D virus (HDV)** | | | |
| Anti-HDV (IgG and IgM) | ☐ Positive<br>☐ Negative<br>☐ Not performed<br>☐ Unknown | | |
| HDV RNA | ☐ Positive<br>☐ Negative<br>☐ Not performed<br>☐ Unknown | | |
| HDV RNA quantity | Copies/ml | IU/ml | log IU/ml |

**5. Diagnosis of hepatitis C virus (HCV)**

| | | | |
|---|---|---|---|
| HCV diagnosis | ☐ Positive, and serological marker results are available<br>☐ Positive, but no serological marker results available<br>☐ Negative<br>☐ Unknown (If unknown, proceed to section 6) | | |
| Anti-HCV | ☐ Positive<br>☐ Negative<br>☐ Not completed<br>☐ Unknown | | |
| HCV RNA | ☐ Positive<br>☐ Negative<br>☐ Not performed<br>☐ Unknown | | |
| HCV RNA quantity | Copies/ml | IU/ml | log IU/ml |
| Received treatment with DAAs | ☐ Yes<br>☐ No<br>☐ Unknown | | |
| If yes, treatment regimen | ☐ Sofosbuvir + Velpatasvir<br>☐ Sofosbuvir + Ledipasvir<br>☐ Sofosbuvir + Daclatasvir<br>☐ Other<br>☐ Unknown | | |
| If other treatment, please specify | | | |
| Date of last treatment (mm/yyyy) | | | |

**6. Other risk factors**

| | | | |
|---|---|---|---|
| Alcohol use | ☐ Yes | ☐ No | ☐ Unknown |
| Hypertension | ☐ Yes | ☐ No | ☐ Unknown |
| Fatty liver disease (steatotic liver) | ☐ Yes | ☐ No | ☐ Unknown |
| Diabetes | ☐ Yes | ☐ No | ☐ Unknown |
| Obesity (BMI > 30) | ☐ Yes | ☐ No | ☐ Unknown |
| Autoimmune hepatitis | ☐ Yes | ☐ No | ☐ Unknown |
| If other risk factor, please specify: | | | |

**7. Referrals**

| | |
|---|---|
| Was the patient referred to another institution? | ☐ Yes<br>☐ No<br>☐ Unknown |

**Table S2: Characteristics of participants with cirrhosis and HCC regarding age, sex, and study site, stratified by study part, Kyrgyzstan, 2024 (n = 1,486)**

| Variable | Cirrhosis | | | | HCC | | | |
|---|---|---|---|---|---|---|---|---|
| | Prospective N = 347 | | Retrospective N = 567 | | Prospective N = 241 | | Retrospective N = 331 | |
| | n | % (95% CI) | n | % (95% CI) | n | % (95% CI) | n | % (95% CI) |
| **Age at medical examination** | | | | | | | | |
| <41 | 60 | 17 (14 – 22) | 104 | 18 (15 – 22) | 7 | 2.9 (1.3 – 6.1) | 9 | 2.7 (1.3 – 5.3) |
| 41-50 | 93 | 27 (22 – 32) | 169 | 30 (26 – 34) | 31 | 13 (9.0 – 18) | 34 | 10 (7.3 – 14) |
| 51-60 | 106 | 31 (26 – 36) | 171 | 30 (27 – 34) | 58 | 24 (19 – 30) | 86 | 26 (21 – 31) |
| 61-70 | 62 | 18 (14 – 22) | 99 | 17 (15 – 21) | 106 | 44 (38 – 51) | 154 | 47 (41 – 52) |
| >70 | 26 | 7.5 (5.2 – 11) | 24 | 4.2 (2.9 – 6.2) | 39 | 16 (12 – 22) | 48 | 15 (11 – 19) |
| **Sex** | | | | | | | | |
| Male | 192 | 55 (50 – 60) | 263 | 46 (42 – 51) | 163 | 68 (61 – 73) | 208 | 63 (57 – 68) |
| Female | 155 | 45 (40 – 50) | 303 | 54 (49 – 58) | 78 | 32 (27 – 39) | 123 | 37 (32 – 43) |
| Missing | 0 | | 1 | | | | | |
| **Study site** | | | | | | | | |
| Bishkek: Gastroenterology Dept. of the National Hospital | 181 | 52 (47 – 57) | 345 | 61 (57 – 65) | 26 | 11 (7.3 – 16) | 5 | 1.5 (0.6 – 3.7) |
| Bishkek: National Centre for Oncology and Haematology | 0 | 0 (0 – 1.1) | 0 | 0 (0 – 0.7) | 86 | 36 (30 – 42) | 158 | 48 (42 – 53) |
| Osh: Osh Interregional United Clinical Hospital | 77 | 22 (18 – 27) | 106 | 19 (16 – 22) | 13 | 5.4 (3.0 – 9.3) | 11 | 3.3 (1.8 – 6.0) |
| Osh: Osh Interregional Oncology Centre | 0 | 0 (0 – 1.1) | 0 | 0 (0 – 0.7) | 80 | 33 (27 – 40) | 78 | 24 (19 – 29) |
| Jalalabad: Regional Clinical Hospital: Therapy Dept. | 89 | 26 (21 – 30) | 116 | 20 (17 – 24) | 1 | 0.4 (0 – 2.7) | 4 | 1.2 (0.4 – 3.3) |
| Jalalabad: Regional Clinical Hospital: Oncology Dept. | 0 | 0 (0 – 1.1) | 0 | 0 (0 – 0.7) | 35 | 15 (10 – 20) | 75 | 23 (18 – 28) |

Participants in retrospective and prospective parts were similar in regards to age (cirrhosis: p = 0.3, HCC: p = 0.8). We observed slightly more male participants in the prospective study part (55%, 95%CI 50 – 60%) compared to the retrospective study part (46%, 95%CI 42 – 51%) among patients with cirrhosis (p = 0.01). Among patients with HCC, we saw a similar tendency (prospective: 68%, 95%CI 61 – 73%, retrospective: 63%, 95%CI 57 – 68%, p = 0.2).

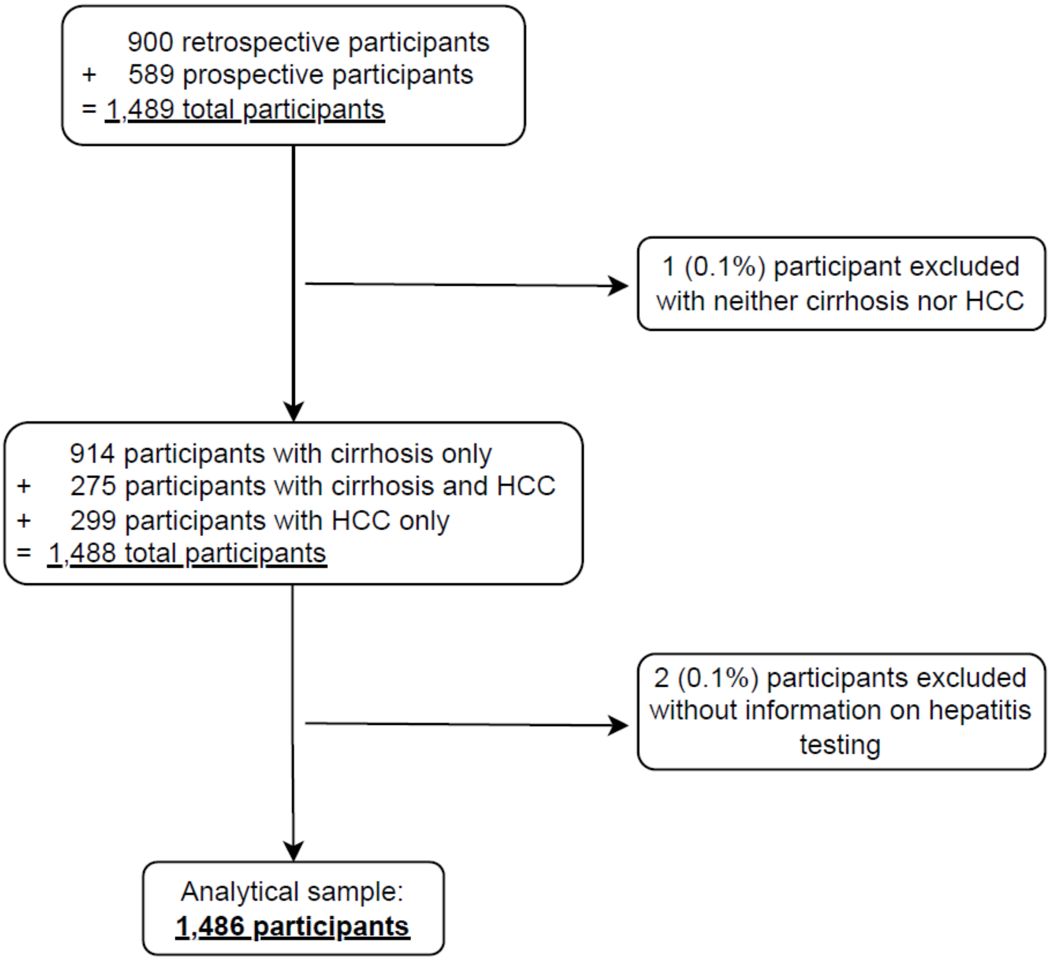


**Figure S1: Study flowchart showing the composition of the study population and excluded participants**

**Table S3: Characteristics of participants with cirrhosis and HCC regarding diagnosis, staging, and outcome, Kyrgyzstan, 2024 (n = 1,486)**

| | Cirrhosis<br>N = 914 | HCC<br>N = 572 |
|---|---|---|
| **First-time diagnosis** | | |
| Yes | 483 (53%) | 491 (86%) |
| No | 428 (47%) | 80 (14%) |
| Missing | 3 | 1 |
| **Cirrhosis stage (according to Child-Pugh)** | | |
| A (5-6) | 104 (12%) | |
| B (7-9) | 626 (69%) | |
| C (10-15) | 173 (19%) | |
| Missing | 11 | |
| **HCC stadium** | | |
| I | | 4 (0.7%) |
| II | | 55 (9.9%) |
| III | | 88 (16%) |
| IV | | 407 (73%) |
| Missing | | 18 |
| **HCC diagnostic method** | | |
| Clinical (laboratory) | | 502 (88%) |
| Instrumental | | 557 (97%) |
| Pathological | | 126 (22%) |
| **Outcome** | | |
| Monitoring | 812 (95%) | 513 (95%) |
| Transplantation | 25 (2.9%) | 11 (2.0%) |
| Death | 16 (1.9%) | 14 (2.6%) |
| Missing | 61 | 34 |

More than half of cirrhosis patients were firstly diagnosed (53%, 483/911) according to their medical record. The majority of participants were in Child stadium B (69%, 626/903), the remaining participants with 19% (173/903) in Child stadium C and 12% (104/903) in Child stadium A. Almost all participants with cirrhosis were in active monitoring (95%, 812/853), while 3% (25/853) had received a liver transplant and 2% (16/853) had died.

At the time of admission to the oncology centre or department, 86% (491/571) of patients with HCC included in the study were diagnosed for the first time. HCC stadium IV was most common with 73% (407/554), followed by stadium III (16%, 88/554), stadium II (10%, 55/554), and stadium I (1%, 4/554). HCC diagnoses were mostly based on instrumental (97%, 557/572) and clinical (88%, 502/572) examinations, and only 22% (126/572) of HCC patients also had a diagnosis based on pathological finding. Most HCC patients were in active monitoring (95%, 513/538), liver transplantation had been received by 2% (11/538), and 3% (14/538) of participants with HCC had died.

**Table S4: Hepatitis diagnosis among participants with cirrhosis and HCC, stratified by study part, Kyrgyzstan, 2024 (n = 1,486)**

| **Variable** | **Cirrhosis** | | | | **HCC** | | | |
|---|---|---|---|---|---|---|---|---|
| | **Prospective N = 347** | | **Retrospective N = 567** | | **Prospective N = 241** | | **Retrospective N = 331** | |
| | n | % (95% CI) | n | % (95% CI) | n | % (95% CI) | n | % (95% CI) |
| **Hepatitis diagnosis** | | | | | | | | |
| Hepatitis B only | 137 | 39 (34 – 45) | 254 | 45 (41 – 49) | 68 | 28 (23 – 34) | 97 | 29 (25 – 35) |
| Hepatitis C only | 79 | 23 (19 – 28) | 122 | 22 (18 – 25) | 72 | 30 (24 – 36) | 94 | 28 (24 – 34) |
| Both hepatitis B and C | 9 | 2.6 (1.3 – 5.0) | 16 | 2.8 (1.7 – 4.6) | 6 | 2.5 (1.0 – 5.6) | 11 | 3.3 (1.8 – 6.0) |
| No hepatitis | 122 | 35 (30 – 40) | 175 | 31 (27 – 35) | 95 | 39 (33 – 46) | 129 | 39 (33 – 44) |

Among participants with cirrhosis, slightly more hepatitis B diagnoses were found in the retrospective study part (45%) compared to the prospective study part (39%), while proportions for hepatitis C diagnosis (22% vs. 23%) and diagnosis with both viruses (2.8% vs. 2.6%) were of similar magnitude. In the prospective study part, slightly more participants had no viral hepatitis diagnosis (35%) than in the retrospective study part (31%). Among participants with HCC, we did not observe substantial differences in proportions for all four viral hepatitis outcome groups between retrospective and prospective study parts: hepatitis B diagnosis (29% vs. 28%), hepatitis C diagnosis (28% vs. 30%), both hepatitis viruses (3.3% vs. 2.5%) and no hepatitis diagnosis (both 39%). We compared the results of the four outcome groups for participants with cirrhosis and HCC, respectively, using Pearson's Chi-Squared test and considered the findings sufficiently comparable (cirrhosis: $p = 0.4$; HCC: $p > 0.9$) to continue analyses with the pooled dataset.

**Table S5: Hepatitis B and C treatment regimens of participants with cirrhosis and HCC, Kyrgyzstan, 2024 (n = 427)**

| **Variable** | **Cirrhosis** | **HCC** | **Overall** |
|---|---|---|---|
| **Hepatitis B treatment regimen** | **N = 242** | **N = 33** | **N = 275** |
| Tenofovir | 159 (66%) | 15 (45%) | 174 (63%) |
| Entecavir | 71 (29%) | 10 (30%) | 81 (29%) |
| Interferon | 0 (0%) | 4 (12%) | 4 (1.5%) |
| Another regimen | 12 (5.0%) | 4 (12%) | 16 (5.8%) |
| **Hepatitis C treatment regimen** | **N = 103** | **N = 49** | **N = 152** |
| Sofosbuvir + Velpatasvir | 52 (50%) | 20 (41%) | 72 (47%) |
| Sofosbuvir + Ledipasvir | 20 (19%) | 6 (12%) | 26 (17%) |
| Sofosbuvir + Daclatasvir | 29 (28%) | 3 (6.1%) | 32 (21%) |
| Another regimen | 2 (1.9%) | 20 (41%) | 22 (14%) |

Most commonly prescribed hepatitis B medications were Tenofovir (63%, 174/275) and Entecavir (29%, 81/275). Only 1% (4/275) of participants received Interferon. For HCV infections, most commonly prescribed combinations were Sofosbuvir + Velpatasvir (47%, 72/152), Sofosbuvir + Daclatasvir (21%, 32/152), and Sofosbuvir + Ledipasvir (17%, 26/152).

**Table S6: Results of mixed-effects multivariable logistic regression with outcome HCC diagnosis among cirrhosis patients, including study sites as random intercept, Kyrgyzstan, 2024 (n = 1,188)**

| **Variable** | **aOR** | **95% CI** | **p-value** |
|---|---|---|---|
| **Hepatitis B diagnosis** | 2.4 | 1.2 – 5.0 | 0.019 |
| **Hepatitis C diagnosis** | 1.6 | 0.76 – 3.4 | 0.2 |
| **Age at medical examination** | | | |
| <41 | 0.08 | 0.01 – 0.52 | 0.008 |
| 41-50 | 0.37 | 0.14 – 0.97 | 0.043 |
| 51-60 | ref. | | |
| 61-70 | 3.0 | 1.5 – 5.8 | 0.001 |
| >70 | 1.6 | 0.46 – 5.6 | 0.5 |
| **Sex** | | | |
| Female | ref. | | |
| Male | 3.8 | 1.9 – 7.4 | <0.001 |
| **Risk factor** | | | |
| Alcohol use | 0.59 | 0.28 – 1.3 | 0.2 |
| Hypertension | 1.8 | 0.94 – 3.5 | 0.076 |
| Steatotic liver | 0.70 | 0.15 – 3.2 | 0.6 |
| Diabetes | 0.74 | 0.28 – 1.9 | 0.5 |
| Obesity (BMI > 30) | 0.82 | 0.36 – 1.9 | 0.6 |
| Autoimmune hepatitis | 1.0 | 0.12 – 8.5 | >0.9 |
| Other risk factor | 0.70 | 0.14 – 3.5 | 0.7 |
