# Supplementary material for: Liver cirrhosis and hepatocellular carcinoma attributable to hepatitis B and C in Kyrgyzstan, 2019–2024: a prospective and retrospective study
Source: Lancet Reg Health Eur. 2026 Mar 28;65:101665. doi: 10.1016/j.lanepe.2026.101665 (PMC13062556; doi:10.1016/j.lanepe.2026.101665)
Supplement: AF_translated_abstract_Russian_notitle [file mmc2.pdf]

This translation in Russian was submitted by the authors and we reproduce it as supplied. It has not been peer reviewed. Our editorial processes have only been applied to the original abstract in English, which should serve as reference for this manuscript.

Предпосылки

Вирусы гепатита B, C и D (HBV/HCV/HDV) могут вызывать хронические инфекции, приводящие к таким осложнениям, как цирроз печени и гепатоцеллюлярная карцинома (ГЦК). Наша цель состояла в оценке доли случаев цирроза и ГЦК, обусловленных HBV и HCV в Кыргызстане.

Методы

Мы собрали информацию о пациентах с диагнозом цирроза и/или ГЦК из медицинских карт клинических больниц и онкологических центров в Бишкеке, Оше и Джалал-Абаде. Исследование включало проспективную часть с 07/2023 по 04/2024 и ретроспективную часть с 01/2019 по 06/2023.

Мы рассчитали этиологическую долю (AF от дословного перевода с англиского «атрибутивная фракция») с 95% доверительным интервалом (95%CI) для HBV и HCV и стратифицировали результаты по возрасту, полу и региону. Мы проанализировали результаты тестов на поверхностный антиген HBV (HBsAg), антитела к HCV (анти-HCV) и HDV (анти-HDV), а также вирусную ДНК/РНК.

Результаты:

Среди участников с циррозом AF для HBV (416/914) составила 46% (95%CI 42-49%) и AF для HCV (226/914) – 25% (95%CI 22-28%). Среди участников с ГЦК AF для HBV (182/572) и HCV (183/572) составили 32% (95%CI 28-36%). Эти расчеты включали 3% пациентов с циррозом (25/914) и ГЦК (17/572), у которых была коинфекция HBV/HCV.

Среди участников, прошедших тестирование на анти-HDV, 78% (306/390) пациентов с циррозом и 50% (44/88) пациентов с ГЦК дали положительный результат на анти-HDV.

Интерпретация:

Большинство случаев цирроза и ГЦК были связаны с HBV и HCV, причем часто встречались коинфекции HBV/HDV. Для предотвращения долгосрочных осложнений и устранения вирусного гепатита как угрозы общественному здоровью в Кыргызстане необходимо внедрить возможность раннего тестирования и лечения.
